# Genome Sequencing of *Microbacterium* sp. Yaish 1, a Bacterial Strain Isolated from the Rhizosphere of Date Palm Trees Affected by Salinity

**DOI:** 10.1128/genomeA.01247-17

**Published:** 2017-11-02

**Authors:** Gerry Aplang Jana, Rashid Al-Yahyai, Mahmoud W. Yaish

**Affiliations:** a Department of Biology, College of Science, Sultan Qaboos University, Muscat, Oman; b Department of Crop Sciences, College of Agricultural and Marine Sciences, Sultan Qaboos University, Muscat, Oman

## Abstract

*Microbacterium* sp. strain Yaish 1 is a rhizospheric bacterium isolated from date palm orchards with high soil salinity. The genome was sequenced, and genes coding for growth-promoting 1-aminocyclopropane-1-carboxylate (ACC) deaminase, siderophore-producing proteins, and tryptophan biosynthesis proteins were identified. Here, we report the draft whole-genome sequencing of the strain.

## GENOME ANNOUNCEMENT

Date palm trees have recently been affected by soil salinity (1, 2), an abiotic stress which can be reduced by plant growth-promoting rhizobacteria (PGPR). PGPR enhance plant survival and development through various mechanisms (3–6). A few of the PGPR directly interact with the plant by forming a symbiotic relationship which enhances biological nitrogen fixation (7). Others synthesize useful enzymes and chemical compounds, such as 1-aminocyclopropane-1-carboxylate (ACC) deaminase, an enzyme that breaks down ACC, which is the main precursor in the biosynthesis of stress-related ethylene phytohormone. This reduces the stress level of plants growing under conditions of high salinity (8, 9). PGPR also produce phytohormones, such as indole acetic acid (IAA), which may enhance root proliferation under abiotic stress conditions (5, 10–13). In addition, some microbes are able to solubilize microelements, such as zinc (14), potassium (15), phosphate (16), and iron ions, which are not readily available for plant uptake (17, 18).

In this work, a semicomplete genome sequence of *Microbacterium* sp. strain Yaish 1, isolated from the saline rhizosphere of date palm trees, was generated to uncover the genes involved in the plant growth-promoting properties of this bacterial strain. The genome was sequenced using Illumina HiSeq 2500 technology at Macrogen, Inc. in South Korea. The paired-end sequencing method was utilized, followed by the Glimmer software (19), which was used to identify the genes within the scaffolds. The genes were annotated using the National Center of Biotechnology (NCBI) Prokaryotic Genome Annotation Pipeline (20).

A total of 31,781,302 reads were generated from the sequencing, which included 23,765,852 mapped paired reads. The assembled results consisted of 3,408,671 bp distributed into only three contigs, with a GC content of about 70.07%. The longest contig was composed of 1,924,248 bp, followed by 1,478,942 bp and 5,481 bp. The reported strain was first identified as *Microbacterium arborescens* based on the sequencing of the 16S rRNA gene and the matrix-assisted laser desorption ionization (MALDI) biotype protein profile analysis. However, subsequent whole-genome sequence analysis revealed that the strain was more similar to *Microbacterium* sp., which was further named *Microbacterium* sp. Yaish 1.

Annotation of the assembled genome resulted in the identification of 3,226 genes and 49 pseudogenes. This included 3,124 genes encoding proteins of known functions. The sequence list included 5 rRNAs, 45 tRNAs, and 3 noncoding RNA genes. The genome analysis revealed the presence of genes that encode siderophore production, ACC deaminase, and a tryptophan biosynthesis coding gene that is important for the production of IAA (21). The presence of these genes within the genome may afford insight into the growth-promoting mechanisms of this bacterial strain.

### Accession number(s).

This whole-genome shotgun project has been deposited at DDBJ/ENA/GenBank under the accession number NPMR00000000. The version described in this paper is the first version, NPMR01000000.
